# Efficacy and safety of a digital therapeutic for alcohol dependence: A multicenter, open‐label, randomized controlled trial

**DOI:** 10.1111/pcn.13874

**Published:** 2025-07-28

**Authors:** Ryuhei So, Kazuhiro Nouso, Sachio Matsushita, Hitoshi Yoshiji, Takefumi Yuzuriha, Eisuke Hida, Hiroki Nishimura, Yusuke Takagi, Yoshinori Horie

**Affiliations:** ^1^ CureApp, Inc. Tokyo Japan; ^2^ Okayama Psychiatric Medical Center Okayama Japan; ^3^ Department of Gastroenterology Okayama City Hospital Okayama Japan; ^4^ National Hospital Organization Kurihama Medical and Addiction Center Yokosuka Japan; ^5^ Department of Gastroenterology Nara Medical University Nara Japan; ^6^ National Hospital Organization Hizen Psychiatric Medical Center Saga Japan; ^7^ Chikugo Yoshii Cocoro Hospital Fukuoka Japan; ^8^ Department of Biostatistics and Data Science The University of Osaka Graduate School of Medicine Osaka Japan; ^9^ Keiai Clinic Tokyo Japan

**Keywords:** alcohol dependence, alcohol use disorder, digital therapeutics, heavy drinking, mobile applications

## Abstract

**Aim:**

Digital therapeutics (DTx) is an emerging treatment modality for enhancing psychosocial interventions *via* software programs, such as smartphone applications (apps). We developed a DTx named ALM‐003 to support treatment for people with alcohol dependence who are pursuing reduced drinking goals. ALM‐003 was designed to promote behavioral change through daily self‐monitoring with personalized feedback, interactive psychoeducational modules, and automated treatment support tools for both patients and physicians. In this multicenter, open‐label, randomized controlled trial (RCT), we aimed to evaluate the efficacy and safety of ALM‐003 in various clinical settings.

**Methods:**

In this RCT, participants with alcohol dependence without serious physical, mental, or social problems due to excessive drinking, and exhibiting high or very high drinking risk levels were randomized into an intervention group (psychosocial intervention enhanced by ALM‐003) or a control group (psychosocial intervention with a basic drinking diary app) for 24 weeks. The primary outcome was the change in the number of heavy drinking days (HDDs) over 28 days (Week 0 to Week 12).

**Results:**

Data analysis of the primary outcome for 136 intervention and 142 control participants was performed. Baseline HDDs were 23.2 and 23.1 days/28 days in the intervention and control groups, respectively. [Correction added on 3 September 2025, after first online publication: The values of the baseline HDDs in the preceding sentence have been corrected from ‘19.4 and 19.1 days’ to ‘23.2 and 23.1 days’.] At Week 12, the intervention and control groups showed a reduction in HDDs of −12.2 and −9.5 days from baseline, with a between‐group difference of −2.79 days/28 days (95% confidence interval: −4.67 to −0.90; *P* = 0.004). Adverse events occurred in 32.9% of participants in the intervention group and 33.6% in the control group. No adverse events were attributed to app use.

**Conclusion:**

ALM‐003 demonstrated efficacy in reducing heavy drinking days among people with alcohol dependence at a high or very high drinking risk.

**Clinical trial registration:**

The trial design was prospectively registered with the Japan Registry of Clinical Trials (jRCT) (https://jrct.niph.go.jp/). The trial identifier is jRCT2032220560.

Reducing the treatment gap and delay for alcohol dependence are crucial challenges in healthcare. The proportion of patients with alcohol dependence receiving specialized treatment is less than one in five worldwide^1^ and less than one in 10 in Japan.^2^ Even among patients receiving treatment, there remains a delay in the first treatment contact after the onset of alcohol‐related problems.^3^, ^4^

To address this challenge, non‐abstinent treatment goals, such as reduced drinking, are introduced.^5^, ^6^ In Japan, 60–75% of people with alcohol dependence prefer reduced drinking goals over abstinence.^2^ Most Japanese addiction specialists consider reduced drinking as a valid intermediate goal.^7^ Reflecting these findings, recent Japanese clinical practice guidelines have described reduced drinking as a viable therapeutic goal for patients without severe physical, mental, or social problems due to excessive alcohol drinking.^8^, ^9^

Delivering treatment in settings accessible for patients could be another strategy to reduce the treatment gap and delay. For instance, 5–9% of patients in primary care clinics^10^, ^11^ and 18.5% in gastroenterology clinics^12^ are suspected to be alcohol‐dependent. General psychiatric clinics are another option because patients with psychiatric disorders frequently have concurrent alcohol use problems.^13^, ^14^

Barriers to treatment delivery in these nonspecialized settings are limited time and resources available for training and the provision of psychosocial interventions.^15^, ^16^ While studies on brief interventions have shown some efficacy for hazardous drinking below the threshold for diagnosing alcohol dependence in nonspecialized settings, evidence for their efficacy in treating alcohol dependence is limited.^17^ A recent trial of internet‐based cognitive behavioral therapy added to primary care treatment for patients with alcohol dependence found small to moderate effects on alcohol consumption at 12 months.^18^ However, the trial included patients with an average daily total alcohol consumption of approximately 40 g, which falls below the threshold for high drinking risk level. To date, interventions using these technologies have not been thoroughly investigated in people with alcohol dependence at high or very high drinking risk.

We, therefore, developed a digital therapeutic (DTx) application (app) for people with alcohol dependence at high or very high drinking risk levels to reduce heavy drinking behaviors. Following the promising results of our pilot randomized controlled trial (RCT),^19^ we conducted this RCT with a larger number of participants and trial sites to evaluate the efficacy and safety of our therapeutic app.

## Methods

### Trial design and settings

This multicenter, open‐label, parallel‐group RCT evaluated the efficacy and safety of the therapeutic app named ALM‐003 which was intended for use in the treatment of people with alcohol dependence exhibiting high or very high drinking risk levels who set a reduced drinking goal.

We conducted this trial at 17 sites, including 13 internal medicine clinics, two general hospital outpatient clinics of the internal medicine departments, and two psychiatric clinics. Participants were recruited from two sources: patients visiting each trial site and those who voluntarily responded to online advertisements. Participants received 7000 JPY (approximately 50 USD) per visit to cover time and travel expenses.

The trial period consisted of two phases: the screening phase (before randomization, Weeks −4 to 0) and the treatment phase (after randomization, Weeks 0 to 24). An overview of the trial schedule is presented in Table S1. A screening phase was set up to exclude people who were considered able to reduce their alcohol consumption without intervention. For this purpose, participants were required to record their daily alcohol consumption during the screening phase using a smartphone app. The app was the same as that used in the control group during the treatment phase.

### Participants

The inclusion criteria for enrolling in the screening phase (Week −4) were as follows: (1) age 20 years or older, (2) diagnosis of alcohol dependence according to the 10th revision of the International Statistical Classification of Diseases and Related Health Problems (ICD‐10) criteria, (3) an average of daily total alcohol consumption exceeding 60 g for men and 40 g for women over the previous 28 days (4) the absence of indications for hospitalization due to alcohol dependence, no serious social and/or familial consequences resulting from alcohol consumption, no life‐threatening organ damage due to alcohol consumption, and the absence of severe alcohol withdrawal symptoms requiring emergency management, (5) a Clinical Institute Withdrawal Assessment for Alcohol‐Revised (CIWA‐Ar) score^20^ of less than 10 at both Week −4 and 0, and (6) daily use of an iPhone with iOS 14.0 or higher or a smartphone with Android version 10 or higher.

The exclusion criteria for the screening phase were: (1) a diagnosis of dementia or intellectual disability, (2) people receiving a structured treatment program for alcohol dependence or for moderating alcohol consumption, or attending a self‐help group program related to alcohol dependence during the 4 weeks prior to Week −4, (3) use of medications for alcohol dependence (disulfiram, cyanamide, acamprosate calcium, and nalmefene), topiramate or any medications not approved in Japan during the 4 weeks prior to Week −4, (4) participation in another intervention trial during the 4 weeks prior to Week −4, (5) participation in the pilot trial of this trial,^19^ (6) exhibiting significant risk of suicide, or a score of 1 or greater on item 9 (item related to suicidal ideation) of the Patient Health Questionnaire 9 (PHQ‐9) at Week −4,^21^, ^22^ (7) pregnant women, breastfeeding women, women who may be pregnant, and women seeking to become pregnant during the duration of the trial, (8) people with no contact details such as permanent email addresses or telephone numbers, and (9) people considered unsuitable to participate in this trial by the trial site investigators.

At randomization (Week 0), participants were included in the treatment phase if they satisfied all of the following criteria: (1) a daily total alcohol consumption during the screening phase (from Week −4 to 0) exceeding 60 g for men and 40 g for women; (2) a CIWA‐Ar score of less than 10; and (3) six or more heavy drinking days (HDDs) during the screening phase (from Week −4 to 0) combined with a failure to achieve five or more consecutive days of abstinence during this period.

### Intervention

Trial physicians were required to complete an e‐learning course on the diagnosis and treatment of alcohol dependence provided by the Japanese Medical Society of Alcohol and Addiction Studies and the Japan Society of Hepatology or an in‐person training program on alcohol dependence for clinicians provided by the Kurihama Medical and Addiction Center. In addition, trial physicians were required to watch a 20‐min tutorial video to understand the app‐enhanced intervention.

Physicians at the trial sites ensured that all randomized participants underwent psychosocial intervention consisting of the recommended components in the Pocket Edition of the Manual of Treatment for Reduced Drinking 1st edition in Japan.^9^ The recommended psychosocial intervention components were: (1) assisting with goal setting in relation to alcohol consumption, (2) monitoring the change in alcohol consumption, (3) monitoring treatment adherence, (4) assessing overall progress, and (5) adjusting treatment goals while respecting participant preferences.

**Fig. 1:**
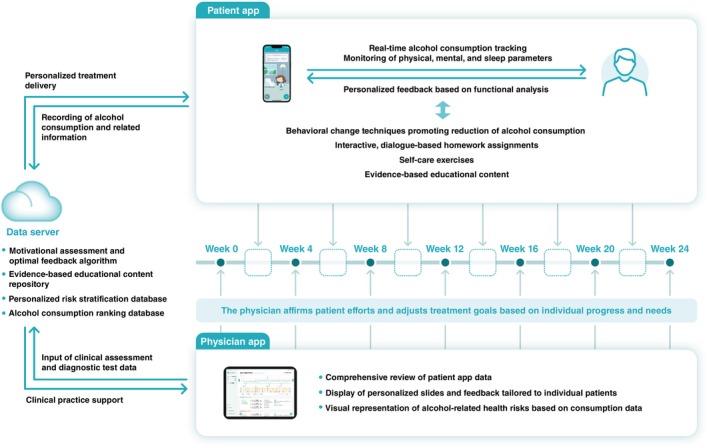
Overview of the app (ALM‐003) for alcohol dependence.

In the intervention group, participants underwent psychosocial intervention enhanced by ALM‐003 (Fig. 1). The ALM‐003 app was designed to help clinicians provide psychosocial intervention and promote behavioral change in people with alcohol dependence who set reduced drinking goals. ALM‐003 consists of a Patient app and a Physician app.

### Patient app

The Patient app enables daily self‐monitoring of alcohol consumption and high‐risk situations that result in heavy drinking, and records daily physical, mental, and sleep status through morning check‐ins and evening check‐outs. These daily tasks typically take only a few minutes to complete. Participants were also encouraged to work on interactive homework programs to understand the chain of drinking behavior, prior triggers, and subsequent short‐ and long‐term consequences, while also learning about strategies to avoid drinking and practicing self‐care skills including mindfulness and stress management techniques. The in‐app assistant, Nancy, poses potentially confrontational questions instead of the physician because a directive or confrontational attitude by physicians may result in poor drinking outcomes.^23^, ^24^ Moreover, Nancy provides personalized feedback, including recommendations for behavioral change techniques and brief information on the physical, mental, and social consequences of drinking.

### Physician app

At each in‐person visit during the treatment phase (Week 0, 4, 8, 12, 16, 20, and 24), the Physician app generated personalized slides to guide the session. These slides reflect the preset content and the patient's input into their Patient app between visits. The slides contained summaries of in‐app medical interviews, self‐monitoring, homework, personalized normative feedback, and functions to set target alcohol consumption levels easily. The personalized normative feedback slide provides a visual representation of alcohol‐related disease risk based on patient alcohol consumption data and ranking of the amount of alcohol consumed compared with the general Japanese population. During each in‐person session, the physician and patient discussed progress toward the target alcohol consumption levels using app‐generated guidance. The physician reviewed the personalized slides with the patient, affirming their efforts, and adjusting the target alcohol consumption level based on patient progress and preferences.

### Control

In the control group, participants recorded their daily alcohol consumption using the same app as in the screening phase, which had a drinking diary function between in‐person sessions. In each in‐person session, clinicians provided the participants with psychosocial intervention according to the pocket manual^9^ without the Physician app but with a booklet for psychoeducation regarding excessive drinking.

Recording daily alcohol consumption in the app and receiving booklet‐based psychoeducation at clinic visits could be considered an enhanced treatment as usual.^25^ However, we included these elements to maintain methodological balance. Using the same drinking diary in both groups could minimize information bias in outcome measurement, while the booklet could compensate for the individualized feedback provided *via* the physician app in the intervention group, thereby keeping clinician contact time and information exposure comparable.

### Baseline characteristics

Patient baseline characteristics and outcome information were collected at Week −4 and 0. Baseline characteristics included basic demographic information and data related to drinking and other addictive behaviors. Regarding drinking behavior‐related data, the following data were collated: age at which the people consumed their first drink, age at the onset of problematic drinking (both as recognized by others and through self‐awareness), history of alcohol dependence treatment, and family history of alcohol problems. Regarding other addictive behavior‐related data, information on smoking history and current smoking status were also collected.

### Efficacy outcomes

The primary outcome was the change in the number of HDDs over 28 days from Week 0 to 12. HDD was defined as a day with an alcohol consumption >60 g for men and > 40 g for women, according to the guidelines of the European Medicines Agency (EMA) on the development of medical products for the treatment of alcohol dependence.^26^ This primary outcome was identical to that used in the Japanese RCT of nalmefene, currently the only approved medication for alcohol reduction treatment in Japan.^27^

The assessment window for HDDs at Week 0 covered Days −28 to −1 when Day 0 was defined as the first day of the treatment phase. Follow‐up assessments of HDDs at Week 12 were based on data collected between the Week 8 and Week 12. Because we allowed a ± 7‐day window for all visits from Week 4 and thereafter, the assessment window at Week 12 varied depending on the actual visit dates. For example, if the Week 8 visit occurred on Day 56 and the Week 12 visit on Day 84, the assessment window was Days 56 to 83. If the visits occurred earlier or later (e.g., Days 49 and 91, or Days 63 and 76), the number of HDDs was adjusted proportionally to reflect a 28‐day period.

We set several secondary outcomes regarding drinking behavior and physical and psychological aspects. We set the change in the number of HDDs from Week 0 to 24 as the secondary outcome. In addition, as for drinking behaviors, the following outcomes were examined at Week 12 and 24: the change in total alcohol consumption (TAC) per 4 weeks from baseline; HDD response (heavy drinking days ≤4 days per 4 weeks); Response Shift Drinking Risk Level (RSDRL); Response Low Drinking Risk Level (RLDRL); 70% decrease in TAC (TAC 70); changes in Non‐Drinking Days per 4 weeks (NDD) from baseline; abstinence; number of days of continuous abstinence; participants who reported improvements in alcohol consumption and alcohol‐related problems using the 7‐point Patient Global Impression of Improvement (PGI‐I) scale; changes in Alcohol Use Disorders Identification Test (AUDIT);^28^, ^29^ and Alcohol Quality of Life Scale (AQoLs).^30^, ^31^

To calculate HDDs and TAC, daily alcohol consumption was measured using the timeline follow‐back method^32^ TLFB is a gold standard to measure alcohol consumption and widely used in phase 3 trials for alcohol dependence. Trial participants review a calendar and retrospectively report their drinking over several preceding weeks. To lessen the recall bias inherent in TLFB, we instructed participants in both groups to record their alcohol consumption each day in the apps. HDDs were calculated at each visit by dividing the number of heavy drinking days by the number of days without missing data over the days prior to each visit, and then multiplying the result by 28. Similarly, TAC was calculated at each time point by dividing the sum of TAC by the number of days without missing data over the days prior to each visit, and then multiplying the result by 28. If the number of days with valid data was less than 12 over 28 days, the corresponding HDDs, TAC, and other outcomes based on alcohol consumption were regarded as missing data.

Secondary outcomes related to biological aspects included changes from baseline in systolic and diastolic blood pressure, body mass index (BMI), and blood test results (Table S1). Non‐fasting blood samples were collected.

In terms of the psychological aspects, we set three secondary outcomes. The PHQ‐9 was used to assess depressive symptoms.^21^, ^22^ The reappraisal subscale of the Emotion Regulation Questionnaire (ERQ)^33^, ^34^ and the savoring the moment subscale of the Savoring Beliefs Inventory (SBI)^35^, ^36^ were used to evaluate how participant coping styles changed.

### Safety outcomes

We collected all adverse events (AEs) and app malfunctions that occurred during the treatment phase.

### Adherence

In the intervention group, we evaluated the number of days for which each participant used the app.

### Randomization, concealment, and blinding

We allocated participants who met the eligibility criteria for the treatment phase equally to either the intervention or control group using stratified block randomization with sex (male or female), WHO Drinking Risk Level (High or Very High), and trial site as stratification factors. Random allocation was automatically performed using an electronic data capture (EDC) system. To ensure allocation concealment, the investigators who enrolled the participants and clinical research coordinators who registered eligible participants with the EDC system did not disclose the block size.

We did not blind participants or clinicians to the allocated arms. We considered that participants would easily realize allocation to the control group when using the smartphone app only with the function of recording daily alcohol consumption. Blinding clinicians also posed difficulties owing to the nature of the intervention, which involved a Patient app and Physician app.

### Sample size

The target sample size was 130 participants for both the intervention and control groups, resulting in a total of 260 participants. We aimed to detect a between‐group difference of 2 HDDs per 4 weeks, informed by RCTs of nalmefene^37^ (95% CI: 0.89 to 2.41 days per 4 weeks). Assuming a standard deviation of 5.7, derived from these RCTs and our pilot RCT^19^, we used a standardized mean difference of 0.35 (2 ÷ 5.7) to calculate the sample size. This conservative estimate reflects assumptions required for the mixed‐effects model for repeated measures (MMRM). We used a two‐sided alpha of 5% and 80% power.

### Statistical analyses

Statistical analyses were performed using SAS software (version 9.4; SAS Institute Inc., Cary, North Carolina, USA). For data visualization, R version 4.4.0 (The R Foundation, Vienna, Austria) with the ggplot2 package was used.^38^

All the efficacy analyses were performed based on the full analysis set (FAS), defined as all randomized participants who provided at least one post‐randomization efficacy assessment.^39^ The FAS is a set that is as close as possible to the intention‐to‐treat principles. The FAS is a group that is as close as possible to the ITT principle. A safety analysis set (SAF) was used to describe adverse events and app malfunctions. The SAF comprised all participants enrolled in the clinical trial and receiving treatment at least once. A safety analysis set (SAF) was used to describe adverse events and app malfunctions. The SAF comprised all participants enrolled in the clinical trial and receiving treatment at least once.

All the efficacy analyses were performed on the full analysis set (FAS), defined as all randomized participants who provided at least one post‐randomization efficacy assessment. The FAS is a set that is as close as possible to the intention‐to‐treat principles. The FAS is a group that is as close as possible to the ITT principle. A safety analysis set (SAF) was used to describe adverse events and app malfunctions. The SAF comprised all participants enrolled in the clinical trial and receiving treatment at least once.

Patient characteristics at baseline are described using mean ± standard deviation (SD) or median for continuous variables, or number (proportion in %) for categorical variables.

We used mixed‐effects models for repeated measures (MMRM) to compare the groups, with adjusted mean changes in HDDs as the primary outcome from Week 0 to 12. Weeks, groups, and their interactions were designated as fixed effects and participants were identified as random effects. Sex, age, and baseline HDD level were incorporated into the model as covariates. Similarly, for secondary outcomes, we calculated and compared the adjusted mean changes in HDDs from Week 0 to 24 and TAC from Week 0 to 12 and 24 using MMRM.

For responder outcomes, including the HDD response and 70% decrease in TAC 70, RSDRL, and RLDRL at Week 12 and 24, the intervention and control groups were compared using the Cochran–Mantel–Haenszel test adjusted for sex and baseline DRL.

For safety outcomes, we calculated the proportion of participants who experienced adverse events and app malfunctions during the trial period. For adherence data, we calculated the average number of days of ALM‐003 use over 28 days in the intervention group.

We conducted this trial following the principles of the Declaration of Helsinki, Japan's Ethical Guidelines for Medical and Health Research Involving Human Subjects, and the International Council for Harmonization's Harmonized Tripartite Guideline for Good Clinical Practice after approval from the institutional review board of Yoyogi Mental Clinic. All participants provided written informed consent before the screening phase after receiving comprehensive information about the trial.

## Results

### Participant flow

**Fig. 2:**
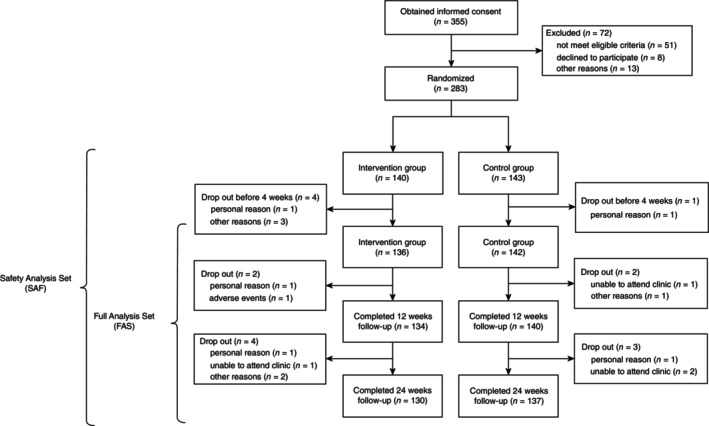
Patient recruitment flowchart.

Recruitment of participants for the screening phase commenced in January 2023. We completed the enrollment and follow‐up of all randomized participants in September 2023. As shown in the participant flowchart (Fig. 2), 283 of the 355 potential participants were randomized. Of the 283 participants who were randomized, 278 were included in the FAS after excluding five who lacked efficacy outcome data after randomization. All 283 participants were included in the SAF for the safety analyses. At Week 12, we could assess the primary outcome in 95.7% (134 of 140) of participants in the intervention group and 97.9% (140 of 143) in the control group. At the final visit (24 weeks), the proportions were 92.9% (130/140) in the intervention group and 95.8% (137/143) in the control group.

### Baseline characteristics

Table 1 shows the baseline demographic and clinical characteristics of the intervention and control groups. Participant mean age was 49.6 (range, 20–73) years, and 36% of the participants were female. At baseline, 55% of the participants were categorized as having a high drinking risk, and the remaining participants were considered to have a very high drinking risk. The baseline characteristics were well balanced between the two groups.

**Table 1:** Baseline characteristics

	Overall	Intervention	Control
Characteristics	*N* = 278*	*N* = 136*	*N* = 142*
Age (years)	49.6 ± 9.8	50.0 ± 10.1	49.2 ± 9.5
Sex (male)	178 (64%)	90 (66%)	88 (62%)
BMI (kg/m^2^)	23.7 ± 3.7	23.7 ± 3.8	23.7 ± 3.6
Lifetime smoking history (present)	164 (59%)	82 (60%)	82 (58%)
Smoking status (current smoker)	61 (22%)	30 (22%)	31 (22%)
Married	195 (70%)	94 (69%)	101 (71%)
Employed	239 (86%)	119 (88%)	120 (85%)
Age at first drink (years)	19.5 ± 2.3	19.7 ± 2.5	19.3 ± 2.0
Age at onset of problem drinking (recognized by others) (years)			
≤39	103 (37%)	52 (38%)	51 (36%)
40–59	152 (55%)	72 (53%)	80 (56%)
≥60	23 (8%)	12 (9%)	11 (8%)
Age at onset of problem drinking (recognized through self‐awareness) (years)			
≤39	125 (45%)	62 (46%)	63 (44%)
40–59	140 (50%)	68 (50%)	72 (51%)
≥60	13 (5%)	6 (4%)	7 (5%)
Previously treated for alcohol dependence	3 (1%)	1 (1%)	2 (1%)
Family history of alcohol problems^†^	37 (13%)	17 (13%)	20 (14%)
HDD^‡^ (days/month)	23.1 ± 4.8	23.2 ± 4.9	23.1 ± 4.7
TAC^§^ (g/day)	88.1 ± 32.0	88.3 ± 30.3	87.9 ± 33.7
WHO DRL			
Low	0 (0%)	0 (0%)	0 (0%)
Medium	0 (0%)	0 (0%)	0 (0%)
High	152 (55%)	74 (54%)	78 (55%)
Very High	126 (45%)	62 (46%)	64 (45%)

* Mean ± SD (standard deviation) or *n*/*N* (%).

^†^ Family history of alcohol problems was assessed using a self‐administered questionnaire including the following question: “Do you have any family members who have had alcohol‐related problems?”.

^‡^ The number of HDDs in the 28 days prior to Week 0, converted to a 28‐day period.

^§^ Average grams of TAC per day over the 28 days prior to Week 0.

BMI, body mass index; DRL, drinking risk level; HDD, heavy drinking days; TAC, total alcohol consumption; WHO, World Health Organization.

### Efficacy outcomes

**Fig. 3:**
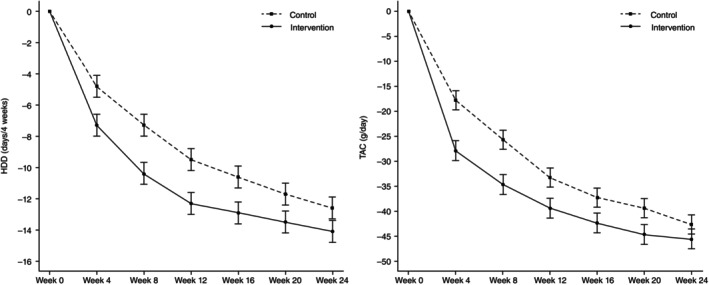
Changes in HDD and TAC from Week 0 to 24. The plots show the adjusted means and standard errors (SE) for Heavy Drinking Days (HDD) and Total Alcohol Consumption (TAC) estimated by the mixed model for repeated measures (MMRM).

Table 2 and Fig. 3 present the alcohol consumption outcomes. Regarding the primary outcome, there was a decrease in the number of HDDs in both groups at Week 12. Specifically, the intervention group showed a reduction of −12.24 ± 0.70 (mean ± standard error [SE]) days, while the control group exhibited a decrease of −9.45 ± 0.68 days (mean ± SE). The adjusted mean difference in the change in HDDs between the two groups was −2.79 days (95% confidence interval [CI]: −4.67 to −0.90), with a significantly greater decrease in the intervention group compared to the control group (*P* = 0.004). Regarding the secondary outcomes based on alcohol consumption, a reduction in HDDs was observed in both groups at Week 24. The intervention group exhibited a decrease of −14.10 ± 0.72 (mean ± SE) days per 4 weeks, while the control group showed a reduction of −12.59 ± 0.70 (mean ± SE) days per 4 weeks. The between‐group difference was −1.5 days (95% CI: −3.4 to 0.40). Details of the results for TAC, HDD response, RSDRL, RLDRL, and TAC 70 are shown in Table 2. The results of other alcohol‐related outcomes are reported in the Supporting Information (Tables S2 and S3).

**Table 2:** Outcomes based on alcohol consumption

				Adjusted Change from Week 0*	Group diff./OR**	
Outcome	Week	Group	Mean ± SD or *n*/*N* (%)	Mean ± SE	(95% CI)	*P*
HDD (days/4 weeks)^†^	Week 0	Intervention	23.2 ± 4.9			
		Control	23.1 ± 4.7			
	Week 12	Intervention	10.5 ± 9.4	−12.2 ± 0.7	−2.8	0.004
		Control	13.5 ± 8.7	−9.5 ± 0.7	(−4.7 to −0.9)	
	Week 24	Intervention	8.6 ± 9.1	−14.1 ± 0.7	−1.5	0.13
		Control	10.4 ± 8.7	−12.6 ± 0.7	(−3.4 to 0.4)	
TAC (g/day)^‡^	Week 0	Intervention	88.3 ± 30.3			
		Control	87.9 ± 33.7			
	Week 12	Intervention	48.4 ± 28.5	−39.4 ± 1.9	−6.1	0.02
		Control	54.3 ± 25.6	−33.3 ± 1.9	(−11.2 to −1.0)	
	Week 24	Intervention	41.9 ± 28.4	−45.6 ± 2.0	−2.9	0.28
		Control	44.8 ± 23.9	−42.7 ± 1.9	(−8.2 to 2.4)	
HDD response^§^	Week 12	Intervention	53/133 (39.8%)		3.1	0.0001
		Control	25/138 (18.1%)		(1.8 to 5.6)	
	Week 24	Intervention	61/128 (47.7%)		2.0	0.01
		Control	43/136 (31.6%)		(1.2 to 3.4)	
RSDRL^§^	Week 12	Intervention	55/133 (41.4%)		2.3	0.002
		Control	32/138 (23.2%)		(1.4 to 3.9)	
	Week 24	Intervention	72/128 (56.3%)		1.7	0.02
		Control	57/136 (41.9%)		(1.1 to 2.8)	
RLDRL^§^	Week 12	Intervention	39/133 (29.3%)		2.2	0.01
		Control	22/138 (15.9%)		(1.2 to 4.1)	
	Week 24	Intervention	51/128 (39.8%)		1.4	0.22
		Control	44/136 (32.4%)		(0.8 to 2.4)	
TAC 70^§^	Week 12	Intervention	22/133 (16.5%)		2.4	0.02
		Control	10/138 (7.2%)		(1.1 to 5.3)	
	Week 24	Intervention	36/128 (28.1%)		2.2	0.01
		Control	20/136 (14.7%)		(1.2 to 4.0)	

* Derived from mixed model repeated measures (MMRM), adjusted mean ± standard error.

** Adjusted mean difference or odds ratio (Mean difference for HDD and TAC; Odds ratio for HDD response, RSDRL, RLDRL and TAC 70).

^†^ Adjusting for sex, age and baseline HDD.

^‡^ Adjusting for sex, age and baseline TAC.

^§^ Adjusting for sex and baseline DRL.

CI, confidence interval; DRL, drinking risk level; HDD, heavy drinking day; RLDRL, response low drinking risk level; RSDRL, response shift drinking risk level; SD, standard deviation; TAC, total alcohol consumption; TAC 70, 70% decrease in total alcohol consumption.

Among biological outcomes, only γ‐GTP exceeded the general reference range at baseline. Mean γ‐GTP values ± SD in the intervention and control groups were 73.6 ± 85.1 U/L and 75.3 ± 88.7 U/L at baseline, 63.5 ± 97.7 U/L and 74.1 ± 109.1 U/L at Week 12, and 65.5 ± 86.5 U/L and 76.8 ± 113.8 U/L at Week 24, respectively. Between‐group differences were − 4.5 (95% CI: −17.3 to 8.4) at Week 12 and − 5.4 (95% CI: −19.5 to 8.7) at Week 24. The results of the other biological outcomes are presented in Table S4.

Regarding psychological outcomes, the mean ± SE scores of PHQ‐9, a screening scale for depressive symptoms, at baseline were below the cutoff of 10 points for screening major depression: 4.3 ± 3.7 in the intervention group and 4.1 ± 4.1 in the control group. The between‐group difference in change was 0.3 (95% CI −0.4 to 0.9). For the subscales of ERQ and SBI, outcomes were used to assess changes in coping styles for difficult situations, and favorable changes were observed in both groups. At 24 weeks, the between‐group differences in change were − 1.4 (95% CI‐2.5 to −0.2) for the reappraisal subscale of the ERQ and −0.2 (95% CI −0.3 to 0.0) for the savoring the moment subscale SBI. Table S5 provides more detailed results of the psychological outcomes.

### Safety outcomes

In this trial, 46 participants (32.9%) in the intervention group and 48 participants (33.6%) in the control group reported experiencing adverse events, none of which were considered related to the apps. One case of pneumonia and one of tendon rupture (both in the intervention group) were reported as serious adverse events. Twenty‐four participants (17.1%) in the intervention group and four (2.8%) in the control group experienced temporary app malfunctions during the trial. None of these malfunctions increased the risk to health.

### Adherence

Among participants who attended trial site visits, none submitted fewer than 12 days of drinking records to the Patient app every 28 days (fewer than 3 days per 7 days), which was the threshold for invalid data in the between‐visit drinking records. App usage was 21.68 ± 6.98 (mean ± SD) days with a median (25th percentile, 75th percentile) of 24.27 (16.77, 28.00) per 4 weeks at Week 12 and 19.84 ± 7.78 (mean ± SD) days with a median (25th percentile, 75th percentile) of 21.37 (13.70, 27.00) per 4 weeks at Week 24.

## Discussion

This trial demonstrates that the therapeutic app intervention may be a novel therapeutic option to achieve alcohol reduction while offering people a favorable balance between efficacy and safety. The app significantly decreased the number of heavy drinking days (HDD) at Week 12, which was the primary outcome. Our intervention also demonstrated superiority over the control group in terms of responder outcomes, such as HDD response, TAC 70%, and RSDRL. The dropout proportion was low (5.65%) in both groups, and no between‐group difference in adverse events was observed.

The mean HDD change in the intervention group in this trial was greater than that in most previous pharmacotherapy trials with comparable baselines, whereas the between‐group differences were similar. In nalmefene trials included in a recent meta‐analysis,^40^ mean HDD changes ranged from −12.5 to −5.6 days per 4 weeks at Week 12, while the intervention group in the current trial showed changes of −12.2 per 4 weeks at Week 12. Meanwhile, the between‐group differences in HDD change were −2.8 days/4 weeks at Week 12, compared to differences ranging from −2.0 to −4.6 days/4 weeks in the nalmefene trials. The larger HDD changes in the intervention group, but comparable between‐group differences in this trial compared with the nalmefene trials, can be attributed to the greater HDD reductions in the control group, which the enhanced treatment as usual was provided as mentioned in the Methods section.^25^ The enhanced control intervention might explain the greater reduction in the control group.^25^

Beyond the primary outcome, our intervention demonstrates clinical significance from multiple perspectives. Responder outcomes adopted in other RCTs, including the HDD response (≤4 HDDs in 4 weeks) and RSDRL (a WHO Drinking Risk Level Reduction by two or more levels), were higher in the intervention group than those in the control group. Previous research has revealed that negative physical, relational, impulsive behavioral, and social consequences of drinking increase significantly with HDDs >3 days per month.^41^ Patients who achieved <3 HDDs per month incurred lower healthcare costs after one year than those with ≥3 days.^42^ Furthermore, WHO DRL reduction has been linked to improvements in psychosocial functioning and is associated with reduced risks of liver disease and cardiovascular disease.^43^, ^44^, ^45^ These findings indicate that improvement in responder outcomes directly contributes to long‐term health and economic benefits.

Although point estimates for all drinking outcomes consistently favored the intervention group, between‐group differences for some outcomes narrowed from Week 12 to Week 24. This attenuation reflected additional improvement in the control group rather than diminishing effects in the intervention group. Such late improvement in the control group was not observed in the RCT and post‐marketing survey of nalmefene.^27^, ^46^ The sustained adherence reminders throughout the 24‐week period, as well as daily app‐based drinking diaries and booklet‐based psychoeducation, may have promoted further improvement in the control group. The incremental long‐term benefit of ALM‐003 should therefore be confirmed in routine clinical practice. The app intervention for alcohol dependence demonstrated a favorable balance between efficacy and safety, as well as minimal training costs. Regarding safety, the nalmefene trial in Japan reported adverse events leading to dropout in 18% of the intervention group.^27^ In the present trial, only 7% of the intervention group dropped out, with no adverse events suspected to be causally related to the app. Additionally, trial site physicians were required only to watch a 20‐min tutorial video, which appears to be a minimal requirement. This combination of efficacy, safety, and ease of implementation highlights the potential of the ALM‐003 intervention as a valuable option for addressing alcohol dependence in various healthcare settings.

Our findings should be interpreted with caution owing to some limitations. The first limitation concerns transportability. We designed ALM‐003 for people with alcohol dependence who aim to reduce their drinking to reduce the treatment gap and delay. Consistent with that purpose, we enrolled a largely treatment‐naïve sample (99%), similar to the population in the Japanese nalmefene RCT.^27^ The efficacy and safety observed in this trial could not be transportable to people with more severe alcohol dependence. Those who experience severe symptoms and repeated relapses should pursue abstinence‐oriented goals rather than reduction as the Japanese guidelines recommend.

Second, the participants and physicians in this trial were not blinded, which may have led to an overestimation of the effect. However, a meta‐epidemiological study revealed no significant difference in effect estimates between trials in which patients, healthcare providers, and outcome assessors were blinded and those without blinding.^47^

Third, as discussed previously, a greater change in outcomes was observed in our control group, possibly due to the effects of daily drinking records using the app together with the sustained adherence reminders throughout the treatment period, compared to previous pill placebo trials with similar participant characteristics.^40^ This indicates that our trial settings may have led to conservative estimates of between‐group differences in the outcomes.

Fourth, our study did not provide evidence clarifying the active ingredients of ALM‐003. For example, we included mindfulness‐related content as a potential active component, aiming to nurture skills such as cognitive reappraisal of emotion and savoring the moment. However, the subscales of the ERQ and the SBI, which were selected as proxies for these skills, did not show greater improvements in the intervention group compared to controls. To identify the therapeutic mechanisms more precisely, future research should employ causal mediation analysis^48^ and trials that randomize individual components of the app.^49^

Fifth, the design of this trial did not allow for a detailed examination of dose–response relationships between app engagement and outcomes. For example, because the level of app use was left to participants' discretion, those who experienced early benefit might have reduced their engagement. Such potential nonlinearity in engagement and outcome warrants further investigation in future trials that randomize the intensity of individual components to better understand the therapeutic mechanisms of the app.

Sixth, we did not measure the workload of either patients or clinicians. The patient app was designed to require only a few minutes of daily input, and the physician app to streamline consultations. To balance contact time and information load, the control group also used the drinking‐diary app and received a standardized psychoeducational booklet. Nevertheless, we collected no empirical data on actual time or effort in either group. The impact of ALM‐003 on patient and clinician workload should therefore be assessed in future implementation studies.

In conclusion, the results demonstrate the favorable efficacy and safety profile of the app‐based intervention for alcohol dependence in people with high or very high drinking risk levels. Future research should determine which subgroups can expect greater benefit from the app and evaluate whether the intervention can help reduce the treatment gap and delay in real‐world settings, thereby building evidence to support broader implementation.

## Funding Information

Japan Agency for Medical Research and Development (AMED) (23he0122018j0003).

## Disclosure statement

RS is an employee at CureApp, Inc., during the conduct of the study; grants from Osake‐no‐Kagaku Foundation, grants from The Mental Health Okamoto Memorial Foundation, grants from Kobayashi Magobe Memorial Medical Foundation, personal fees from Otsuka Pharmaceutical Co., Ltd., Nippon Shinyaku Co., Ltd., Takeda Pharmaceutical Co., Ltd., and Sumitomo Pharma Co., Ltd. outside the submitted work; In addition, RS has a patent JP2022049590A, US20220084673A1 pending, a patent JP2022178215A pending, a patent JP2022070086 pending, and a patent JP2023074128A pending.

YH, KN, SM, HY and TY received personal fees from CureApp, Inc. for serving as trial coordinators. YH, KN, HY and TY received personal fees from Otsuka Pharmaceutical. SM has received a research grant from Asahi Quality & Innovations, Ltd. and has received speaker's honoraria from EA Pharma, Otsuka Pharmaceutical, Yoshitomi Yakuhin, Eisai, Nippon Shinyaku, Otsuka Pharmaceutical and MSD.

EH received personal fees from CureApp, Inc. for serving as a trial statistician.

HN and YT are employees at CureApp, Inc., during the conduct of the study.

## Author contributions

RS led the development of ALM‐003, which was the digital therapeutic app evaluated in this trial. RS conceptualized the trial and drafted the initial methodology. KN, SM, HY, TY, and YH reviewed the methodology and coordinated the trial implementation. EH contributed as the trial statistician. RS and HN prepared the first draft of the manuscript. All authors contributed to data interpretation and provided critical review and revision of the manuscript draft. All authors read and approved the final manuscript for submission.

## Supporting information


**Table S1.** Measurements and Assessments at Each Visit
**Table S2.** Abstinence‐oriented outcomes
**Table S3.** Alcohol‐related outcomes
**Table S4.** Biological outcomes
**Table S5.** Psychological Outcomes

**Data S1** Supporting information

## Data Availability

The data that support the findings of this study are available from the corresponding author, RS, upon reasonable request. However, some restrictions may apply due to ethical considerations, legal agreements, and/or commercial sensitivity.
