# Supplementary material for: Efficacy and safety of a digital therapeutic for alcohol dependence: A multicenter, open‐label, randomized controlled trial
Source: Psychiatry Clin Neurosci. 2025 Jul 28;79(10):667–76. doi: 10.1111/pcn.13874 (PMC12498122; doi:10.1111/pcn.13874)
Supplement: Supplementary file 1 — Table S1. Measurements and Assessments at Each Visit Table S2. Abstinence‐oriented outcomes Table S3. Alcohol‐related outcomes Table S4. Biological outcomes Table S5. Psychological Outcomes [file PCN-79-667-s002.docx]

**Table S1 Measurements and Assessments at Each Visit**

| **Measurement Assessment Evaluation** | | **Screening Phase** | | | | **Treatment Phase** | | | | **Withdrawn Patients** |
| --- | --- | --- | --- | --- | --- | --- | --- | --- | --- | --- |
|  |  | **Week**  **-4** | **Week 0** | **Week 4** | **Week 8** | **Week 12** | **Week 16** | **Week 20** | **Week 24** |  |
|  |  | **visit 1** | **visit 2** | **visit 3** | **visit 4** | **visit 5** | **visit 6** | **visit 7** | **visit 8** |  |
| Informed consent | | ● |  |  |  |  |  |  |  |  |
| Baseline characteristics | | ● |  |  |  |  |  |  |  |  |
| Eligibility check | | ● | ● |  |  |  |  |  |  |  |
| Randomization | |  | ● |  |  |  |  |  |  |  |
| Height | | ● |  |  |  |  |  |  |  |  |
| Weight | | ● | ● | ● | ● | ● | ● | ● | ● | ● |
| BMI | | ● | ● | ● | ● | ● | ● | ● | ● | ● |
| Blood Pressure | | ● | ● | ● | ● | ● | ● | ● | ● | ● |
| Blood Test* | | ● |  |  |  | ● |  |  | ● | ● |
| Pregnancy Test | | ● |  |  |  |  |  |  |  |  |
| Observer-reported/rated | TLFB |  | ● | ● | ● | ● | ● | ● | ● | ● |
|  | CIWA-Ar | ● | ● | ● | ● | ● | ● | ● | ● |  |
|  | Adverse event monitoring | ● | ● | ● | ● | ● | ● | ● | ● | ● |
| Participant-reported/rated | AUDIT |  | ● |  |  | ● |  |  | ● |  |
|  | AQoLs |  | ● |  |  | ● |  |  | ● |  |
|  | PHQ-9 | ● |  |  |  | ● |  |  | ● |  |
|  | SBI |  | ● |  |  | ● |  |  | ● |  |
|  | ERQ |  | ● |  |  | ● |  |  | ● |  |
|  | PGI-I |  |  | ● | ● | ● | ● | ● | ● |  |

BMI: body mass index; TLFB: timeline follow-back; CIWA-Ar: Clinical Institute Withdrawal Assessment for Alcohol, Revised; AUDIT: Alcohol Use Disorders Identification Test; AQoL: Alcohol Quality of Life Scale; PHQ-9: Patient Health Questionnaire-9; SBI: Savoring Beliefs Inventory; ERQ: Emotion Regulation Questionnaire; PGI-I: Patient Global Impression of Improvement Scale.

*Blood tests included WBC, RBC, Hb, Ht, PLT, MCV, MCH, MCHC, AST, ALT, γ-GTP, ALB, T-Bil, UA, T-Chol, HDL-Chol, LDL-Chol, and TG.

**Table S2 Abstinence-oriented outcomes**

| **Outcome** | **Week** | **Group** | **n/N (%) or LS Mean ± SE*** | **Group difference**** |  |
| --- | --- | --- | --- | --- | --- |
|  |  |  |  | **(95% CI)** | **p** |
| NDD (days/4 weeks) † | Week 0 | Intervention | 1.44 ± 2.55 |  |  |
|  |  | Control | 1.25 ± 2.18 |  |  |
|  | Week 12 | Intervention | 5.17 ± 6.08 | 3.68 ± 0.43 | 0.2 |
|  |  | Control | 4.82 ± 5.16 | 3.48 ± 0.42 | (-1.0 to 1.4) |
|  | Week 24 | Intervention | 6.42 ± 6.78 | 4.99 ± 0.43 | 0.1 |
|  |  | Control | 6.25 ± 6.18 | 4.93 ± 0.42 | (-1.1 to 1.2) |
| Abstinence rate‡ | Week 12 | Intervention | 5 / 133 (3.8%) | 4.7 | 0.10 |
|  |  | Control | 1 / 138 (0.7%) | (0.6 to 37.8) |  |
|  | Week 24 | Intervention | 3 / 128 (2.3%) | 1.6 | 0.61 |
|  |  | Control | 2 / 136 (1.5%) | (0.3 to 9.2) |  |
| Number of days of continuous abstinence† | Week 12 | Intervention  (n=5) | 17.4 ± 3.6 | 9.2 | 0.59 |
|  |  | Control  (n=1) | 8.1 ± 10.8 | (-149.0 to 167.5) |  |
|  | Week 24 | Intervention  (n=3) | 13.0 | - | - |
|  |  | Control  (n=1) | 15.0 | - |  |

*Least squares mean ± standard error

**Odds ratio or adjusted mean difference

†Adjusting for gender, age baseline NDD

‡Adjusting for gender and baseline DRL

CI, confidence interval; DRL, drinking risk level; NDD, nondrinking days.

**Table S3 Alcohol-related outcomes**

| **Outcome** | **Week** | **Group** | **Mean ± SD** | **Adjusted Change from Week 0*** | **Group difference**** |  |
| --- | --- | --- | --- | --- | --- | --- |
|  |  |  |  | **LS Mean ± SE** | **(95% CI)** | **p** |
| AUDIT† | Week 0 | Intervention | 19.0 ± 5.2 |  |  |  |
|  |  | Control | 19.2 ± 6.1 |  |  |  |
|  | Week 12 | Intervention | 14.4 ± 5.9 | -4.5 ± 0.4 | -0.3 | 0.56 |
|  |  | Control | 14.9 ± 5.8 | -4.2 ± 0.4 | (-1.4 to 0.8) |  |
|  | Week 24 | Intervention | 12.9 ± 6.0 | -5.9 ± 0.4 | -0.4 | 0.55 |
|  |  | Control | 13.5 ± 6.3 | -5.5 ± 0.4 | (-1.6 to 0.9) |  |
| AQoLs‡ | Week 0 | Intervention | 11.3 ± 11.2 |  |  |  |
|  |  | Control | 12.7 ± 13.1 |  |  |  |
|  | Week 12 | Intervention | 7.7 ± 8.4 | -3.7 ± 0.5 | 0.9 | 0.19 |
|  |  | Control | 7.6 ± 9.0 | -4.7 ± 0.5 | (-0.5 to 2.4) |  |
|  | Week 24 | Intervention | 5.4 ± 6.7 | -6.2 ± 0.5 | 0.2 | 0.74 |
|  |  | Control | 5.8 ± 7.3 | -6.4 ± 0.5 | (-1.1 to 1.5) |  |
| PGI-I§ alcohol consumption | Week 12 | Intervention |  | 2.7 ± 0.1 | -0.2 | 0.08 |
|  |  | Control |  | 2.9 ± 0.1 | (-0.4 to -0.0) |  |
|  | Week 24 | Intervention |  | 2.3 ± 0.1 | -0.2 | 0.15 |
|  |  | Control |  | 2.3 ± 0.1 | (-0.4 to 0.1) |  |
| PGI-I§ alcohol-related problems | Week 12 | Intervention |  | 3.0 ± 0.1 | -0.2 | 0.17 |
|  |  | Control |  | 3.2 ± 0.1 | (-0.4 to 0.1) |  |
|  | Week 24 | Intervention |  | 2.7 ± 0.1 | -0.1 | 0.25 |
|  |  | Control |  | 2.8 ± 0.1 | (-0.4 to 0.1) |  |

*Least squares mean ± standard error

**Adjusted mean difference

†Adjusting for gender, age and baseline AUDIT

‡Adjusting for gender, age and baseline AQoLs

§Adjusting for gender, age and baseline TAC

SD, standard deviation; CI, confidence interval; TAC, total alcohol consumption. AUDIT (Alcohol Use Disorders Identification Test): Higher scores indicate a greater risk of hazardous and harmful alcohol use. AQoLs (Alcohol Quality of Life Scale): Lower scores suggest better alcohol-related quality of life. Patient Global Impression of Improvement (PGI-I): Lower scores indicate greater patient-reported improvement in alcohol consumption and alcohol-related problems.

**Table S4 Biological outcomes**

| **Outcome** | **Week** | **Group** | **Mean ± SD** | **Adjusted Change from Week 0*** | **Group difference**** |  |
| --- | --- | --- | --- | --- | --- | --- |
|  |  |  |  | **LS Mean ± SE** | **(95% CI)** | **p** |
| WBC† | Week 0 | Intervention | 6127.9 ± 1634.0 |  |  |  |
|  |  | Control | 5844.2 ± 1551.9 |  |  |  |
|  | Week 12 | Intervention | 5711.7 ± 1405.8 | -297.0 ± 95.3 | 1.0 | 0.99 |
|  |  | Control | 5563.1 ± 1611.1 | -298.0 ± 93.6 | (-262.6 to 264.5) |  |
|  | Week 24 | Intervention | 5844.6 ± 1420.5 | -169.9 ± 103.6 | 85.3 | 0.56 |
|  |  | Control | 5604.6 ± 1745.7 | -255.1 ± 100.5 | (-199.3 to 369.9) |  |
| RBC† | Week 0 | Intervention | 455.8 ± 48.4 |  |  |  |
|  |  | Control | 445.6 ± 50.4 |  |  |  |
|  | Week 12 | Intervention | 451.0 ± 46.8 | -3.5 ± 2.1 | 2.4 | 0.42 |
|  |  | Control | 439.6 ± 51.5 | -5.9 ± 2.0 | (-3.4 to 8.1) |  |
|  | Week 24 | Intervention | 447.4 ± 43.2 | -7.0 ± 2.2 | -0.5 | 0.88 |
|  |  | Control | 438.9 ± 51.9 | -6.5 ± 2.1 | (-6.5 to 5.6) |  |
| Hb† | Week 0 | Intervention | 14.4 ± 1.4 |  |  |  |
|  |  | Control | 14.2 ± 1.4 |  |  |  |
|  | Week 12 | Intervention | 14.1 ± 1.4 | -0.3 ± 0.1 | 0.0 | 0.79 |
|  |  | Control | 13.9 ± 1.4 | -0.3 ± 0.1 | (-0.2 to 0.2) |  |
|  | Week 24 | Intervention | 14.0 ± 1.4 | -0.4 ± 0.1 | -0.1 | 0.62 |
|  |  | Control | 13.9 ± 1.4 | -0.4 ± 0.1 | (-0.2 to 0.1) |  |
| Ht† | Week 0 | Intervention | 43.4 ± 3.9 |  |  |  |
|  |  | Control | 42.8 ± 3.9 |  |  |  |
|  | Week 12 | Intervention | 43.1 ± 4.0 | -0.2 ± 0.2 | 0.1 | 0.68 |
|  |  | Control | 42.4 ± 3.8 | -0.3 ± 0.2 | (-0.4 to 0.7) |  |
|  | Week 24 | Intervention | 42.8 ± 3.8 | -0.5 ± 0.2 | -0.1 | 0.63 |
|  |  | Control | 42.4 ± 3.8 | -0.4 ± 0.2 | (-0.7 to 0.4) |  |
| PLT† | Week 0 | Intervention | 26.3 ± 6.1 |  |  |  |
|  |  | Control | 26.1 ± 5.8 |  |  |  |
|  | Week 12 | Intervention | 25.8 ± 5.8 | -0.5 ± 0.3 | -0.04 | 0.92 |
|  |  | Control | 25.7 ± 5.5 | -0.5 ± 0.3 | (-0.7 to 0.7) |  |
|  | Week 24 | Intervention | 25.4 ± 5.7 | -1.0 ± 0.3 | 0.1 | 0.88 |
|  |  | Control | 25.0 ± 5.5 | -1.1 ± 0.3 | (-0.7 to 0.8) |  |
| MCV† | Week 0 | Intervention | 95.4 ± 4.8 |  |  |  |
|  |  | Control | 96.5 ± 6.6 |  |  |  |
|  | Week 12 | Intervention | 95.8 ± 4.8 | 0.3 ± 0.3 | -0.4 | 0.28 |
|  |  | Control | 97.0 ± 6.9 | 0.7 ± 0.3 | (-1.1 to 0.3) |  |
|  | Week 24 | Intervention | 95.8 ± 4.9 | 0.2 ± 0.3 | -0.5 | 0.31 |
|  |  | Control | 97.1 ± 6.7 | 0.6 ± 0.3 | (-1.4 to 0.4) |  |
| MCH† | Week 0 | Intervention | 31.7 ± 1.8 |  |  |  |
|  |  | Control | 32.1 ± 2.5 |  |  |  |
|  | Week 12 | Intervention | 31.3 ± 1.8 | -0.4 ± 0.1 | -0.1 | 0.25 |
|  |  | Control | 31.8 ± 2.7 | -0.2 ± 0.1 | (-0.4 to 0.1) |  |
|  | Week 24 | Intervention | 31.3 ± 1.9 | -0.4 ± 0.1 | -0.1 | 0.53 |
|  |  | Control | 31.7 ± 2.4 | -0.3 ± 0.1 | (-0.4 to 0.2) |  |
| MCHC† | Week 0 | Intervention | 33.2 ± 1.0 |  |  |  |
|  |  | Control | 33.2 ± 1.0 |  |  |  |
|  | Week 12 | Intervention | 32.7 ± 1.0 | -0.5 ± 0.1 | -0.1 | 0.59 |
|  |  | Control | 32.8 ± 1.1 | -0.4 ± 0.1 | (-0.3 to 0.1) |  |
|  | Week 24 | Intervention | 32.7 ± 1.1 | -0.5 ± 0.1 | 0.0 | 0.98 |
|  |  | Control | 32.7 ± 1.2 | -0.5 ± 0.1 | (-0.2 to 0.2) |  |
| AST† | Week 0 | Intervention | 26.9 ± 12.6 |  |  |  |
|  |  | Control | 28.6 ± 19.4 |  |  |  |
|  | Week 12 | Intervention | 25.3 ± 9.8 | -1.1 ± 0.8 | -1.9 | 0.10 |
|  |  | Control | 28.9 ± 21.0 | 0.8 ± 0.8 | (-4.2 to 0.4) |  |
|  | Week 24 | Intervention | 26.7 ± 13.7 | 0.2 ± 1.4 | -3.9 | 0.04 |
|  |  | Control | 32.2 ± 25.7 | 4.0 ± 1.3 | (-7.6 to -0.1) |  |
| ALT† | Week 0 | Intervention | 23.6 ± 13.1 |  |  |  |
|  |  | Control | 25.1 ± 18.5 |  |  |  |
|  | Week 12 | Intervention | 22.9 ± 14.0 | -0.3 ± 0.9 | -0.9 | 0.50 |
|  |  | Control | 25.0 ± 18.3 | 0.5 ± 0.9 | (-3.5 to 1.7) |  |
|  | Week 24 | Intervention | 23.7 ± 15.8 | 0.4 ± 1.4 | -2.8 | 0.16 |
|  |  | Control | 28.0 ± 26.3 | 3.2 ± 1.4 | (-6.7 to 1.1) |  |
| γ-GTP† | Week 0 | Intervention | 73.6 ± 85.1 |  |  |  |
|  |  | Control | 75.3 ± 88.7 |  |  |  |
|  | Week 12 | Intervention | 63.5 ± 97.7 | -4.7 ± 4.6 | -4.5 | 0.49 |
|  |  | Control | 74.1 ± 109.1 | -0.2 ± 4.6 | (-17.3 to 8.4) |  |
|  | Week 24 | Intervention | 65.5 ± 86.5 | -4.0 ± 5.1 | -5.4 | 0.45 |
|  |  | Control | 76.8 ± 113.8 | 1.4 ± 5.0 | (-19.5 to 8.7) |  |
| ALB† | Week 0 | Intervention | 4.5 ± 0.3 |  |  |  |
|  |  | Control | 4.5 ± 0.3 |  |  |  |
|  | Week 12 | Intervention | 4.4 ± 0.3 | -0.1 ± 0.0 | 0 | 0.60 |
|  |  | Control | 4.4 ± 0.3 | -0.1 ± 0.0 | (-0.04 to 0.1) |  |
|  | Week 24 | Intervention | 4.4 ± 0.3 | -0.1 ± 0.0 | -0.03 | 0.46 |
|  |  | Control | 4.5 ± 0.4 | -0.03 ± 0.0 | (-0.1 to 0.1) |  |
| T-Bil† | Week 0 | Intervention | 0.7 ± 0.3 |  |  |  |
|  |  | Control | 0.6 ± 0.3 |  |  |  |
|  | Week 12 | Intervention | 0.7 ± 0.3 | 0.04 ± 0.0 | -0.03 | 0.66 |
|  |  | Control | 0.7 ± 0.3 | 0.1 ± 0.0 | (-0.1 to 0.1) |  |
|  | Week 24 | Intervention | 0.7 ± 0.3 | 0.03 ± 0.0 | -0.1 | 0.03 |
|  |  | Control | 0.7 ± 0.3 | 0.1 ± 0.0 | (-0.1 to -0.01) |  |
| UA† | Week 0 | Intervention | 5.9 ± 1.5 |  |  |  |
|  |  | Control | 5.7 ± 1.3 |  |  |  |
|  | Week 12 | Intervention | 6.0 ± 1.6 | 0.1 ± 0.1 | -0.2 | 0.18 |
|  |  | Control | 5.9 ± 1.4 | 0.2 ± 0.1 | (-0.4 to 0.1) |  |
|  | Week 24 | Intervention | 6.1 ± 1.5 | 0.2 ± 0.1 | -0.01 | 0.91 |
|  |  | Control | 5.9 ± 1.3 | 0.2 ± 0.1 | (-0.2 to 0.2) |  |
| T-chol† | Week 0 | Intervention | 215.0 ± 36.4 |  |  |  |
|  |  | Control | 214.8 ± 37.3 |  |  |  |
|  | Week 12 | Intervention | 211.1 ± 34.1 | -4.7 ± 1.9 | 0.1 | 0.96 |
|  |  | Control | 209.6 ± 36.6 | -4.8 ± 1.9 | (-5.2 to 5.4) |  |
|  | Week 24 | Intervention | 205.8 ± 31.8 | -9.4 ± 2.1 | 0.2 | 0.94 |
|  |  | Control | 205.3 ± 36.4 | -9.6 ± 2.0 | (-5.4 to 5.9) |  |
| HDL-chol† | Week 0 | Intervention | 75.7 ± 22.9 |  |  |  |
|  |  | Control | 76.3 ± 22.3 |  |  |  |
|  | Week 12 | Intervention | 72.8 ± 21.7 | -3.2 ± 1.0 | -1.0 | 0.50 |
|  |  | Control | 74.8 ± 22.3 | -2.2 ± 1.0 | (-3.7 to 1.8) |  |
|  | Week 24 | Intervention | 70.6 ± 21.4 | -5.1 ± 1.1 | -1.6 | 0.27 |
|  |  | Control | 72.7 ± 22.5 | -3.4 ± 1.0 | (-4.5 to 1.3) |  |
| LDL-chol† | Week 0 | Intervention | 110.9 ± 31.5 |  |  |  |
|  |  | Control | 109.5 ± 33.9 |  |  |  |
|  | Week 12 | Intervention | 112.5 ± 30.1 | 1.0 ± 1.6 | 2.3 | 0.29 |
|  |  | Control | 107.8 ± 29.7 | -1.3 ± 1.6 | (-2.0 to 6.7) |  |
|  | Week 24 | Intervention | 106.0 ± 27.5 | -5.3 ± 1.8 | -0.7 | 0.79 |
|  |  | Control | 105.1 ± 30.7 | -4.6 ± 1.7 | (-5.5 to 4.2) |  |
| TG† | Week 0 | Intervention | 166.0 ± 126.9 |  |  |  |
|  |  | Control | 172.0 ± 147.2 |  |  |  |
|  | Week 12 | Intervention | 153.3 ± 122.3 | -11.7 ± 10.0 | -12.4 | 0.38 |
|  |  | Control | 167.5 ± 172.3 | 0.6 ± 9.8 | (-40.1 to 15.3) |  |
|  | Week 24 | Intervention | 171.0 ± 154.2 | 5.3 ± 11.9 | 10.2 | 0.54 |
|  |  | Control | 167.4 ± 168.9 | -4.9 ± 11.5 | (-22.5 to 42.9) |  |
| SBP† | Week 0 | Intervention | 129.0 ± 16.7 |  |  |  |
|  |  | Control | 129.4 ± 14.7 |  |  |  |
|  | Week 12 | Intervention | 125.3 ± 15.7 | -4.4 ± 1.0 | 2.0 | 0.16 |
|  |  | Control | 123.6 ± 13.9 | -6.3 ± 1.0 | (-0.8 to 4.7) |  |
|  | Week 24 | Intervention | 123.3 ± 15.7 | -7.1 ± 1.0 | -1.6 | 0.26 |
|  |  | Control | 124.6 ± 13.3 | -5.4 ± 1.0 | (-4.4 to 1.2) |  |
| DBP† | Week 0 | Intervention | 81.4 ± 12.5 |  |  |  |
|  |  | Control | 82.7 ± 10.5 |  |  |  |
|  | Week 12 | Intervention | 79.4 ± 11.7 | -2.6 ± 0.7 | 1 | 0.31 |
|  |  | Control | 79.1 ± 10.2 | -3.6 ± 0.7 | (-1.0 to 3.0) |  |
|  | Week 24 | Intervention | 78.3 ± 10.6 | -4.2 ± 0.7 | -0.8 | 0.45 |
|  |  | Control | 79.4 ± 9.6 | -3.4 ± 0.7 | (-2.8 to 1.2) |  |
| BMI† | Week 0 | Intervention | 23.7 ± 3.8 |  |  |  |
|  |  | Control | 23.7 ± 3.6 |  |  |  |
|  | Week 12 | Intervention | 23.4 ± 3.7 | -0.1 ± 0.1 | 0.1 | 0.19 |
|  |  | Control | 23.4 ± 3.7 | -0.2 ± 0.1 | (-0.1 to 0.3) |  |
|  | Week 24 | Intervention | 23.3 ± 3.8 | -0.3 ± 0.1 | 0.1 | 0.35 |
|  |  | Control | 23.3 ± 3.7 | -0.4 ± 0.1 | (-0.1 to 0.2) |  |

*Least squares mean ± standard error

**Adjusted mean difference

†Adjusting for sex, age, and baseline values of each item

SD, standard deviation; CI, confidence interval; WBC, White Blood Cell Count; RBC, Red Blood Cell Count; Hb, Hemoglobin; Ht, Hematocrit; PLT, Platelet Count; MCV, Mean Corpuscular Volume; MCH, Mean Corpuscular Hemoglobin; MCHC, Mean Corpuscular Hemoglobin Concentration; AST, Aspartate Aminotransferase; ALT, Alanine Aminotransferase; γ-GTP, Gamma-Glutamyl Transpeptidase; ALB, Albumin; T-Bil, Total Bilirubin; UA, Uric Acid; T-chol, Total Cholesterol; HDL-chol, High-Density Lipoprotein Cholesterol; LDL-chol, Low-Density Lipoprotein Cholesterol; TG, Triglycerides.

**Table S5 Psychological Outcomes**

| Outcome | Week | Group | Mean ± SD | Adjusted Change from Week 0* | Group difference** |  |
| --- | --- | --- | --- | --- | --- | --- |
|  |  |  |  | LS Mean ± SE | (95% CI) | p |
| PHQ-9† | Week 0 | Intervention | 4.3 ± 3.7 |  |  |  |
|  |  | Control | 4.1 ± 4.1 |  |  |  |
|  | Week 12 | Intervention | 3.4 ± 2.9 | -0.8 ± 0.2 | 0.3 | 0.38 |
|  |  | Control | 3.0 ± 3.8 | -1.1 ± 0.2 | (-0.4 to 0.9) |  |
|  | Week 24 | Intervention | 2.6 ± 2.4 | -1.6 ± 0.2 | 0.1 | 0.59 |
|  |  | Control | 2.3 ± 2.4 | -1.7 ± 0.2 | (-0.4 to 0.7) |  |
| The reappraisal subscale of the ERQ‡ | Week 0 | Intervention | 27.6 ± 5.6 |  |  |  |
|  |  | Control | 27.7 ± 5.7 |  |  |  |
|  | Week 12 | Intervention | 28.8 ± 5.4 | 1.1 ± 0.4 | -0.2 | 0.79 |
|  |  | Control | 29.0 ± 5.8 | 1.3 ± 0.4 | (-1.3 to 1.0) |  |
|  | Week 24 | Intervention | 29.4 ± 5.6 | 1.7 ± 0.4 | -1.4 | 0.02 |
|  |  | Control | 30.7 ± 5.7 | 3.0 ± 0.4 | (-2.5 to -0.2) |  |
| The savoring the moment subscale of the SBI§ | Week 0 | Intervention | 1.3 ± 1.0 |  |  |  |
|  |  | Control | 1.5 ± 1.0 |  |  |  |
|  | Week 12 | Intervention | 1.3 ± 1.0 | 0.0 ± 0.1 | -0.2 | 0.02 |
|  |  | Control | 1.7 ± 0.9 | 0.2 ± 0.1 | (-0.4 to -0.04) |  |
|  | Week 24 | Intervention | 1.5 ± 0.9 | 0.3 ± 0.1 | -0.2 | 0.04 |
|  |  | Control | 1.9 ± 0.9 | 0.4 ± 0.1 | (-0.3 to -0.01) |  |

*Least squares mean ± standard error

**Adjusted mean difference

†Adjusting for sex age and baseline PHQ-9

‡Adjusting for sex, age and baseline ERQ

§Adjusting for sex, age and baseline SBI

SD, standard deviation; CI, confidence interval. PHQ-9 (Patient Health Questionnaire-9): Higher scores indicated more severe depressive symptoms. ERQ (Emotion Regulation Questionnaire): Higher scores suggest greater use of specific emotion regulation strategies. SBI (Savoring Belief Inventory): Higher scores indicate stronger capacity to fully enjoy experiences while they are occurring.
