# Supplementary material for: Efficacy and safety of a digital therapeutic for alcohol dependence: A multicenter, open‐label, randomized controlled trial
Source: Psychiatry Clin Neurosci. 2025 Jul 28;79(10):667–76. doi: 10.1111/pcn.13874 (PMC12498122; doi:10.1111/pcn.13874)
Supplement: Supplementary file 2 — Data S1 Supporting information [file PCN-79-667-s001.docx]

**CONSORT 2010 checklist of information to include when reporting a randomised trial**

| **Section/Topic** | **Item No** | **Checklist item** | **Reported on page No** |  |
| --- | --- | --- | --- | --- |
| **Title and abstract** | | | | |
|  | 1a | Identification as a randomised trial in the title | Page: 1 | **Title** Efficacy and Safety of a Digital Therapeutic for Alcohol Dependence: A Multi-Center, Open-Label, Randomized Controlled Trial |
|  | 1b | Structured summary of trial design, methods, results, and conclusions (for specific guidance see CONSORT for abstracts) | Page: 1 | **Abstract  Aims**  Digital therapeutics is an emerging treatment modality for enhancing psychosocial interventions via software programs, such as smartphone applications (apps)… **Methods** In this multi-center, open-label, randomized controlled trial, participants with alcohol dependence without serious physical, mental, or social problems ... **Results** Data analysis of the primary outcome for 136 intervention and 142 control participants was performed... **Conclusion** ALM-003 demonstrated efficacy and safety in reducing heavy drinking days among people with alcohol dependence at a high or very high drinking risk. |
| **Introduction** | | | | |
| Background and objectives | 2a | Scientific background and explanation of rationale | Page: 3-4 | **Introduction** Alcohol dependence is attributable to 71% of the burden of alcohol-related deaths...  Non-abstinent treatment goals, such as reduced drinking, are recognized as a new strategy to increase early treatment uptake for alcohol dependence… People with alcohol dependence are reluctant to seek treatment in specialized care settings.... To address these unmet needs, new possibilities for intervention using mobile devices have been studied... |
|  | 2b | Specific objectives or hypotheses | Page: 4 | Following the promising results of a pilot randomized controlled trial (RCT),23 we conducted this pivotal RCT with a larger number of participants and trial sites to confirm the efficacy and safety of our therapeutic app. |
| **Methods** | | | | |
| Trial design | 3a | Description of trial design (such as parallel, factorial) including allocation ratio | Page: 4, 8 | **Trial design and settings** This multicenter, open-label, parallel-group RCT evaluated the efficacy and safety of the therapeutic app named ALM-003…  **Randomization, concealment, and blinding** Participants who met the eligibility criteria for the treatment phase were allocated equally to either the intervention group or the control group ... |
|  | 3b | Important changes to methods after trial commencement (such as eligibility criteria), with reasons | N/A | No changes to methods reported after trial commencement |
| Participants | 4a | Eligibility criteria for participants | Page: 4-5 | **Participants** The inclusion criteria for enrolling in the screening phase (Week -4) were as follows: (1) aged 20 years or older, (2) diagnosis of alcohol dependence according to ICD-10 criteria… |
|  | 4b | Settings and locations where the data were collected | Page: 4 | **Trial design and settings** The trial was conducted at 17 sites, including 13 internal medicine clinics, two general hospital outpatient clinics of the internal medicine departments, and two psychiatric clinics… |
| Interventions | 5 | The interventions for each group with sufficient details to allow replication, including how and when they were actually administered | Page: 5-6  Figure 1 | **Intervention** Physicians at the trial sites ensured that all randomized participants underwent psychosocial intervention consisting of the recommended components... **Patient app** The Patient app enables daily self-monitoring of alcohol consumption and high-risk situations that result in heavy drinking, and records daily physical, mental, and sleep status... **Physician app** At each in-person visit during the treatment phase (Week 0, 4, 8, 12, 16, 20, and 24), the Physician app generated personalized slides to guide the session...  **Control** In the control group, participants recorded their daily alcohol consumption using the same app as in the screening phase... |
| Outcomes | 6a | Completely defined pre-specified primary and secondary outcome measures, including how and when they were assessed | Page: 7-8 | **Efficacy outcomes** The primary outcome was the change in the number of HDDs over 28 days from Week 0 to 12… **Safety outcomes** We collected all adverse events (AEs) and app malfunctions that occurred during the treatment phase. **Adherence** In the intervention group, we evaluated the number of days for which each participant used the app. |
|  | 6b | Any changes to trial outcomes after the trial commenced, with reasons | N/A |  |
| Sample size | 7a | How sample size was determined | Page: 8 | **Sample size** The target sample size was 130 participants for both the intervention and control groups, resulting in a total of 260 participants. The sample size was calculated based on the primary outcome measures of the HDDs. We assumed a standardized mean difference of 0.35, with a two-sided significance level of 5% and a statistical power of 80%. |
|  | 7b | When applicable, explanation of any interim analyses and stopping guidelines | N/A |  |
| Randomisation: |  |  |  |  |
| Sequence generation | 8a | Method used to generate the random allocation sequence | Page: 8 | Random allocation was automatically performed using an electronic data capture (EDC) system. |
|  | 8b | Type of randomisation; details of any restriction (such as blocking and block size) | Page: 8 | stratified block randomization with sex (male or female), WHO Drinking Risk Level (High or Very High), and trial site as stratification factors. |
| Allocation concealment mechanism | 9 | Mechanism used to implement the random allocation sequence (such as sequentially numbered containers), describing any steps taken to conceal the sequence until interventions were assigned | Page: 8 | To ensure allocation concealment, the investigators who enrolled the participants and clinical research coordinators who registered eligible participants with the EDC system did not disclose the block size. |
| Implementation | 10 | Who generated the random allocation sequence, who enrolled participants, and who assigned participants to interventions | Page: 8 | the investigators who enrolled the participants and clinical research coordinators who registered eligible participants with the EDC system did not disclose the block size. |
| Blinding | 11a | If done, who was blinded after assignment to interventions (for example, participants, care providers, those assessing outcomes) and how | Page: 8 | We did not blind participants or clinicians to the allocated arms. We considered that participants, would easily realize allocation to the control group when using the smartphone app only with the function of recording daily alcohol consumption. Blinding clinicians also posed difficulties owing to the nature of the intervention, which involved a Patient app and Physician app. |
|  | 11b | If relevant, description of the similarity of interventions | Page: 5-6 | Physicians at the trial sites ensured that all randomized participants underwent psychosocial intervention consisting of the recommended components in the Pocket Edition of the Manual of Treatment for Reduced Drinking 1st edition in Japan.10 The recommended psychosocial intervention components were: (1) assisting with goal setting in relation to alcohol consumption, (2) monitoring the change in alcohol consumption, (3) monitoring treatment adherence, (4) assessing overall progress, and (5) adjusting treatment goals while respecting participant preferences. In the intervention group, participants underwent psychosocial intervention enhanced by ALM-003 (Figure 1)....  **Control** In the control group, participants recorded their daily alcohol consumption using the same app as in the screening phase, which only had a drinking diary function between in-person sessions... |
| Statistical methods | 12a | Statistical methods used to compare groups for primary and secondary outcomes | Page: 8-9 | **Statistical analyses** We used mixed-effects models for repeated measures (MMRM) to compare the groups, with adjusted mean changes in HDDs as the primary outcome from Week 0 to 12... |
|  | 12b | Methods for additional analyses, such as subgroup analyses and adjusted analyses | N/A | No subgroup analyses or adjusted analyses were pre-specified in the methods |
| **Results** | | | | |
| Participant flow (a diagram is strongly recommended) | 13a | For each group, the numbers of participants who were randomly assigned, received intended treatment, and were analysed for the primary outcome | Page: 9 Figure 2 | **Participant flow** As shown in the participant flowchart (Figure 2), 283 of the 355 potential participants were randomized... |
|  | 13b | For each group, losses and exclusions after randomisation, together with reasons | Page: 9 Figure 2 | **Participant flow** The follow-up rates for the primary outcome (Week 12) were 95.7% (134/140) and 97.9% (140/143) in the intervention and control groups, respectively. At the final visit (24 weeks), the rates were 92.9% (130/140) and 95.8 % (137/143) in the intervention and control groups, respectively. |
| Recruitment | 14a | Dates defining the periods of recruitment and follow-up | Page: 9 | **Participant flow** Recruitment of participants for the screening phase commenced in January 2023. We completed the enrollment and follow-up of all randomized participants in September 2023. |
|  | 14b | Why the trial ended or was stopped | N/A | Trial completed as planned |
| Baseline data | 15 | A table showing baseline demographic and clinical characteristics for each group | Page: 10 Table 1 | **Baseline characteristics** Table 1 shows the baseline demographic and clinical characteristics of the intervention and control groups... |
| Numbers analysed | 16 | For each group, number of participants (denominator) included in each analysis and whether the analysis was by original assigned groups | Page: 9 Table 1 | **Participant flow** Of the 283 participants who were randomized, 278 were included in the FAS after excluding five people who lacked efficacy outcome data after randomization. All 283 participants were included in the SAF for safety analyses. The follow-up rates for the primary outcome (Week 12) were 95.7% (134/140) and 97.9% (140/143) in the intervention and control groups, respectively. |
| Outcomes and estimation | 17a | For each primary and secondary outcome, results for each group, and the estimated effect size and its precision (such as 95% confidence interval) | Page: 10-11 Table 2, Figure 3, Table S2-S5 | **Efficacy outcomes** Table 2 and Figure 3 present the alcohol consumption outcomes. Regarding the primary outcome, there was a decrease in the number of HDDs in both groups at Week 12... |
|  | 17b | For binary outcomes, presentation of both absolute and relative effect sizes is recommended | Table 2 | Table 2 |
| Ancillary analyses | 18 | Results of any other analyses performed, including subgroup analyses and adjusted analyses, distinguishing pre-specified from exploratory | N/A | No results of subgroup analyses or adjusted analyses were reported |
| Harms | 19 | All important harms or unintended effects in each group (for specific guidance see CONSORT for harms) | Page: 11 | **Safety outcomes** In this trial, 46 participants (32.9%) in the intervention group and 48 participants (33.6%) in the control group reported experiencing adverse events, none of which were considered related to the apps... |
| **Discussion** | | | | |
| Limitations | 20 | Trial limitations, addressing sources of potential bias, imprecision, and, if relevant, multiplicity of analyses | Page: 12 | Our findings should be interpreted with caution owing to some limitations... |
| Generalisability | 21 | Generalisability (external validity, applicability) of the trial findings | Page: 12 | The efficacy and safety results shown in this trial are not applicable to people with more severe forms of alcohol dependence characterized by a complete loss of control over drinking, resulting in an inability to reduce alcohol consumption.  In conclusion, the results demonstrate the favorable efficacy and safety profile of app-based interventions for alcohol dependence in people with high or very high drinking risk levels... |
| Interpretation | 22 | Interpretation consistent with results, balancing benefits and harms, and considering other relevant evidence | Page: 12 | The app intervention for alcohol dependence demonstrated a favorable balance between efficacy and safety, as well as minimal training costs...  Digital therapeutics, such as ALM-003, have the potential to contribute to alcohol-related harm reduction by providing a more accessible and effective treatment for alcohol dependence. |
| Other information | | | | |
| Registration | 23 | Registration number and name of trial registry | Page: 1 | The trial design was prospectively registered with the Japan Registry of Clinical Trials (jRCT). The trial identifier is jRCT2032220560. |
| Protocol | 24 | Where the full trial protocol can be accessed, if available | N/A | Protocol access information not provided |
| Funding | 25 | Sources of funding and other support (such as supply of drugs), role of funders |  | Japan Agency for Medical Research and Development (AMED) (23he0122018j0003) |
